# Quantitative assessment of atelectasis formation under high frequency jet ventilation during liver tumour ablation–A computer tomography study

**DOI:** 10.1371/journal.pone.0282724

**Published:** 2023-04-03

**Authors:** Karolina Galmén, Jan G. Jakobsson, Gaetano Perchiazzi, Jacob Freedman, Piotr Harbut

**Affiliations:** 1 Department of Anaesthesiology, Danderyd University Hospital, Stockholm, Sweden; 2 Department of Surgical Sciences, Hedenstierna Laboratory, Uppsala University, Uppsala, Sweden; 3 Department of Anaesthesia, Operation and Intensive Care, Uppsala University Hospital, Uppsala, Sweden; 4 Department of Surgery and Urology, Danderyd University Hospital, Stockholm, Sweden; 5 Division of Clinical Sciences, Karolinska Institutet at Danderyd University Hospital, Stockholm, Sweden; Sant Anna Hospital: Clinica Sant’Anna, SWITZERLAND

## Abstract

**Background:**

High frequency jet ventilation (HFJV) can be used to minimise sub-diaphragmal organ displacements. Treated patients are in a supine position, under general anaesthesia and fully muscle relaxed. These are factors that are known to contribute to the formation of atelectasis. The HFJV-catheter is inserted freely inside the endotracheal tube and the system is therefore open to atmospheric pressure.

**Aim:**

The aim of this study was to assess the formation of atelectasis over time during HFJV in patients undergoing liver tumour ablation under general anaesthesia.

**Method:**

In this observational study twenty-five patients were studied. Repeated computed tomography (CT) scans were taken at the start of HFJV and every 15 minutes thereafter up until 45 minutes. From the CT images, four lung compartments were defined: hyperinflated, normoinflated, poorly inflated and atelectatic areas. The extension of each lung compartment was expressed as a percentage of the total lung area.

**Result:**

Atelectasis at 30 minutes, 7.9% (SD 3.5, p = 0.002) and at 45 minutes 8,1% (SD 5.2, p = 0.024), was significantly higher compared to baseline 5.6% (SD 2.5). The amount of normoinflated lung volumes were unchanged over the period studied. Only a few minor perioperative respiratory adverse events were noted.

**Conclusion:**

Atelectasis during HFJV in stereotactic liver tumour ablation increased over the first 45 minutes but tended to stabilise with no impact on normoinflated lung volume. Using HFJV during stereotactic liver ablation is safe regarding formation of atelectasis.

## Introduction

### Background

Ablation of liver tumours is a growing field in minimally invasive oncologic surgery [1, 2]. Percutaneous ablations with computer assisted techniques, using microwave or irreversible electroporation has decreased the local recurrence rate within six months from 30% to near 10% [2]. Safe and effective ablation procedures are dependent on fixed position of the target organ. Respiratory movements of the diaphragm and associated displacement of solid organs cause targeting problems, especially during CT guided, stereotactic procedures [3–5]. Ablative treatments of liver tumours are commonly done under general anaesthesia and conventional tidal volume mechanical ventilation moves organs adjacent to the diaphragm, potentially jeopardizing the efficacy of the ablation [3, 6]. Reduction of respiration related movements can be achieved by using high frequency jet ventilation (HFJV) and total intravenous anaesthesia with profound neuromuscular block [3, 7]. High frequency jet ventilation is a well-established method used since the 1960’s. It has mostly been used for ear-nose-and throat-surgery where the anatomical space is shared by the surgeon and the anaesthetist. HFJV uses very high breathing frequencies, usually 110-400/min, and low tidal volumes. The tidal volumes are below dead-space and therefore other mechanisms are involved in the gas exchange compared to conventional ventilation [8]. The use of HFJV for the purpose of minimising breathing related organ movement is relatively new.

### What is known?

Formation of atelectasis during general anaesthesia in supine position is a well-known phenomenon [9, 10] and occurs in approximately 90% of anesthetised patients [9]. Poorly aerated, atelectatic areas in the lungs are perfused and gas exchange is thus impaired [11]. It is a multifactorial process caused by absorption of alveolar gas, compression of the lung tissue leading to decreased compliance and reduced functional residual capacity (FRC). This has been robustly explored during conventional ventilation [12–17], and is known to contribute to postoperative complications as well as increased health care costs [18]. Several studies have also used CT-imaging to investigate how to minimise atelectasis formation during anaesthesia and have concluded that applying positive end expiratory pressure (PEEP) is an essential factor [15, 19, 20]. How lung atelectasis evolves over time during HFJV is not well explored. There is one early study on dogs, from the 1980’s, where no signs of atelectasis were found after HFJV. Another study including eight ICU patients with ALI (acute lung injury) where superimposed HFJV resulted in rapid alveolar recruitment of dependent lung areas when studied with computed tomography [21–23]. There are obvious multiple risk factors for formation of atelectasis during anaesthesia and HFJV. Lung ventilation is performed in an system open to atmospheric pressure, thus application of conventional PEEP is not possible and also the patient is positioned in a supine position with full muscle relaxation, which are all factors facilitating the occurrence of atelectasis.

### Aim

The primary aim was to quantify atelectasis during HFJV using CT-imaging. A secondary aim was to assess the frequency and extent of perioperative respiratory adverse events.

## Methods

### Ethics

The study conforms to the standards of the Declaration of Helsinki and was approved by the Regional Ethics Committee in Stockholm (Dnr 2016/1124-32, June 7^th^, 2016) as well as by the local Radiation Protection Committee at Danderyd University Hospital (Project number 2016–1, June 1^st^, 2016). Written informed consent for participation and publication was obtained from all patients. The study is registered at Clinicaltrials.gov, protocol ID 2017/1158-32.

### Study setup and inclusion

This is a prospective, observational study. Patients were included after being selected for liver tumour ablation with a stereotactic technique. Twenty-five consecutive patients were included. The exclusion criteria were (i) any presence of severe, poorly controlled lung disease for ventilatory reasons and (ii) patients below the age of 50 years for radiation reasons.

### Anaesthesia technique

All patients had total intravenous anesthesia with propofol 10 mg/ml (Propofol-Lipuro®, B. Braun Melsungen AG, Melsungen, Germany) and remifentanil 50 mikrgr/ml (Ultiva®, GlaxoSmithKline AB, Solna, Sweden). Dosages were according to clinical practice and standards for modus TCI. This means, for propofol, after the induction dose, a TCI dose between 2–5 mg/kg/min and for remifentanil a maintenance TCI-dose of 2–10 ng/ml. Neuromuscular block was achieved by rocuronium (Esmeron®, MSD, Haarlem, Netherlands). An initial dose before intubation according to clinical practice of approximately 0.6 mg/kg and thereafter incremental doses when needed, directed by train-of-four (TOF) monitoring.

All patients were monitored with standard ECG, SpO_2_ and invasive blood pressure. Trans-cutaneous CO_2_ (tcCO_2_) was measured with Radiometer TCM5 (Triolab AB, Mölndal, Sweden). Train-of-four was used to monitor muscle relaxant (Philips, Philips Medizin Systeme, Boeblingen GmbH, Hewlett-Packard-Str. 2, 71034 Boeblingen, Germany).

### Ventilation

HFJV has been routine for this type of surgery at our hospital since the start in 2010. It is also used in other centres for the same purpose. HFJV keeps the target organs almost immobilised and therefor creating good surgical conditions. All patients were intubated with an endotracheal tube, a size 9 for men and size 8 for women, as local routine to enhance CO_2_ washout. Once the endotracheal intubation had been performed, a first contrast enhanced CT-scan series of the thorax and liver was done. HFJV was initiated just before the first CT-scan acquisition. This was done by disconnecting the endotracheal tube from the conventional ventilator and inserting a thin jet cannula (Laserjet 40, double lumen jet catheter acc Biro, Acutronic Medical System AG, Hirzel, Germany) through the endotracheal tube. A Monsoon III ventilator was used during anaesthesia with HFJV (Acutronic, Switzerland). Fixed settings for driving pressure (DP) 1.2–1.4 bar, oxygen supplementation FiO2 0.8, frequency 220 min^-1^ and I/E (Inspiration duration ID) ratio 40% were used. Safety limits were set by the operator for pause pressure (PP) and peak inspiratory pressure (PIP). Humidification with sterile water through the jet-catheter was used during the whole procedure to avoid drying and damage to the tracheal mucosa.

### Method of CT/radiology and image analysis

All patients were placed in the supine position in the computed tomography machine (Toshiba Aquilion One). Scans from the base of the lungs were repeated every 15 minutes up until 45 minutes into the procedure, which gave a total of four scans for each patient. The lung programme in the CT software was specifically set up for this study to get as low radiation dose as possible with acceptable quality for analysis. For the CT-images used within the study protocol, the exposure was a volume of 10 cm at 50 mA and 120 kV, with a slice thickness of 0,5 mm.

Before the first CT-scan was performed, all patients received intravenous contrast as a part of the routine procedure for this type of surgery. This could partly overlap the HU of atelectasis and is further discussed in the limitation section.

In some cases, the time for lung scanning (every 15 minutes) coincided with the treatment scanning of the liver and lung bases for the ablation procedure. When this was the case, these scans were used for analysis, with exposure at 140 mA and 120 kV, the slice thickness being 1 mm. All scans were analysed at the level of one cm above the right diaphragm.

In the CT scans, lung parenchyma was manually outlined by tracing the border between the pleura and the surrounding tissues. This was performed by two of the authors, one with previous experience of the method. The results of the tracing were then reviewed by an experienced radiologist (MB). The CT images were further analysed using scripts for the Image Processing Toolbox for MATLAB R2020 (MATLAB, The MathWorks, Natick, MA, USA), purposely written by one of the authors (G.P.). In this way it was possible to define, in accordance with previous convention [24, 25], four lung compartments: hyperinflated (HU between − 1000 and − 900), normoinflated (HU between − 900 and − 500), poorly inflated (HU between − 500 and − 100) and noninflated or atelectatic (HU between − 100 and + 100). The extension of each lung compartment was expressed as percentage of the total lung area in each scan.

The study was terminated as the ablation procedure was completed. All patients were thereafter extubated and transferred to the post anaesthesia care unit (PACU).

Perioperative respiratory adverse events were registered and analysed. These included oxygen desaturations, carbon dioxide retention, pneumothorax and respiratory support need postoperatively as well as prolonged PACU stay.

### Primary and secondary endpoints

The primary aim was to study the quantity of atelectasis formation using CT-imaging, during the initial 45 minutes of the ablation procedure with HFJV. A secondary aim was to assess the frequency and extent of perioperative respiratory adverse events.

### Sample size calculation

The magnitude of atelectasis formation under HFJV is unknown. An exploratory sample of 25 unique subjects was proposed and accepted.

### Statistics

This is an observational study. Data was tested for normality with the Shapiro Wilks test. Descriptive statistics were used for demographic statistics, mean and SD, median and range as applicable. Repeated Measures ANOVA was used on data that was normally distributed and the Friedmans test was used on non-normally distributed data. Tukeys test was used for pairwise multiple comparisons. A p<0.05 was considered statistically significant. All statistical tests were conducted with SigmaPlot (version 14, Software Inc, San Jose, California, USA).

## Results

Twenty-five patients were included. One patient was excluded as no computed tomography scan over the lungs was performed, thus 24 patients’ data sets were analysed. Patient characteristics are presented in Table 1.

**Table 1:** Patient characteristics presented as mean (SD) or median (IQR) as applicable.

Variables		Overall series
Age	Median (years)	70.5 (61–76)
Sex	Male n (%)	16 (67)
	Female n (%)	8 (33)
BMI	Median (kg m^-2^)	25 (24–30)
ASA-score	ASA 1 (%)	1 (4)
	ASA 2 (%)	5 (21)
	ASA 3 (%)	18 (75)
Smoking	Yes (%)	2 (8)
	No (%)	22 (92)*
Lung disease	Yes (%)	6 (25)**
	No (%)	18(75)

* 8 patients with previous smoking habits

** 2 patients with mild asthma, 2 patients with lung metastasis, 1 patient with pulmonary hypertension and Sjogren’s disease, 1 patient with earlier postoperative pulmonary embolism.

### Patient characteristics

Mean time on HFJV for all patients was 79 minutes (SD 29). Seven patients had HFJV beyond the 45-minute protocol.

### CT-scans for analysis of atelectasis

83 scans were included in the analysis. Four patients had baseline and one additional scan at 15 minutes. Five patients had baseline and two additional scans at 15 and 30 minutes. Fifteen patients had baseline CT and the additional scans at 15, 30 and 45 minutes. The first baseline CT-scan was taken at 11.9 (SD 6.9) minutes after the initiation of HFJV.

**Fig 1:**
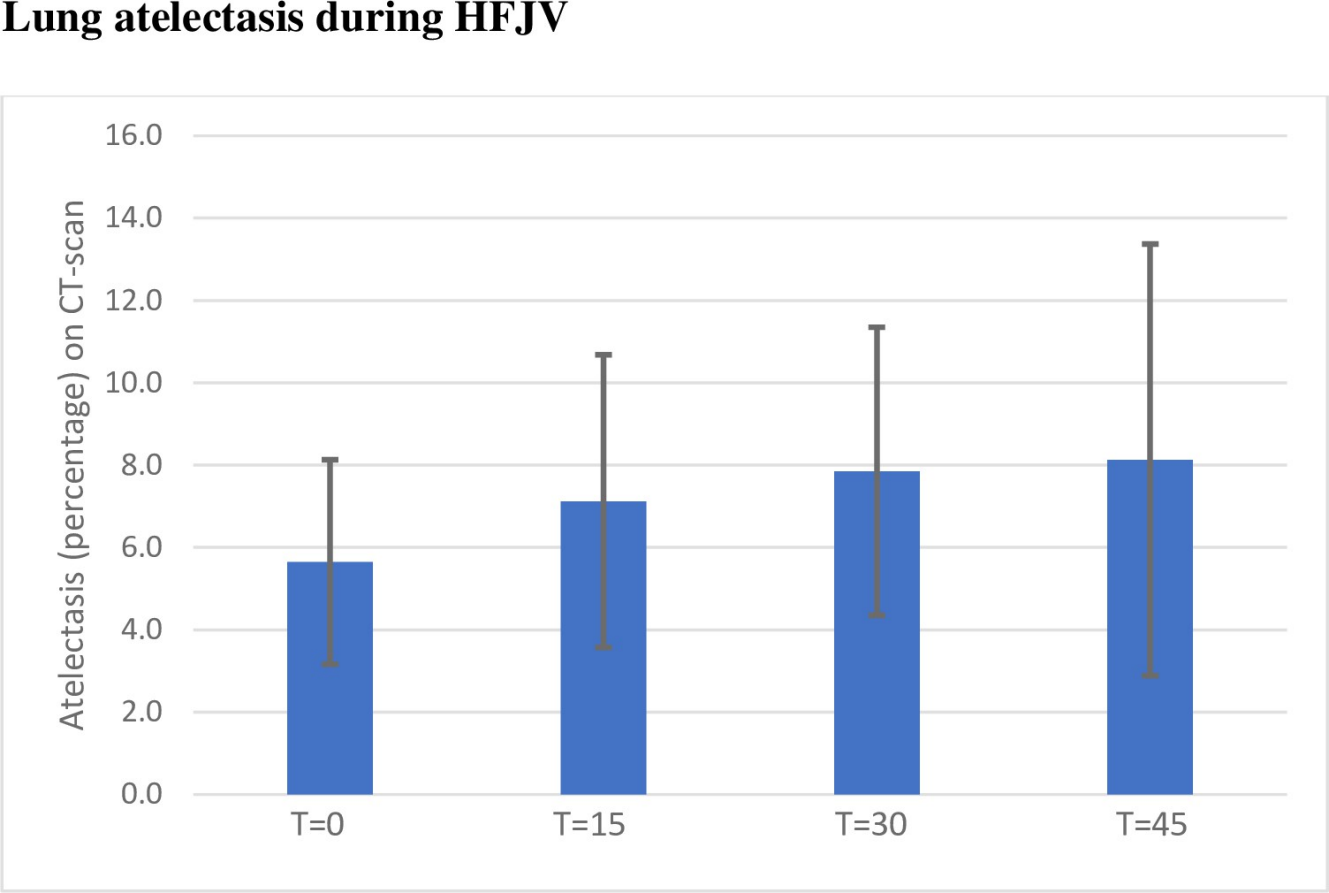
Mean atelectasis in percentage (HU -100-+100) and standard deviation bars. T = 0 being the first CT after initiation of HFJV (high frequency jet ventilation) and thereafter CT-scans every 15 minutes. A significant raise in the amount of atelectasis could be seen at timepoints T = 30 and T = 45 when compared to baseline.

**Fig 2:**
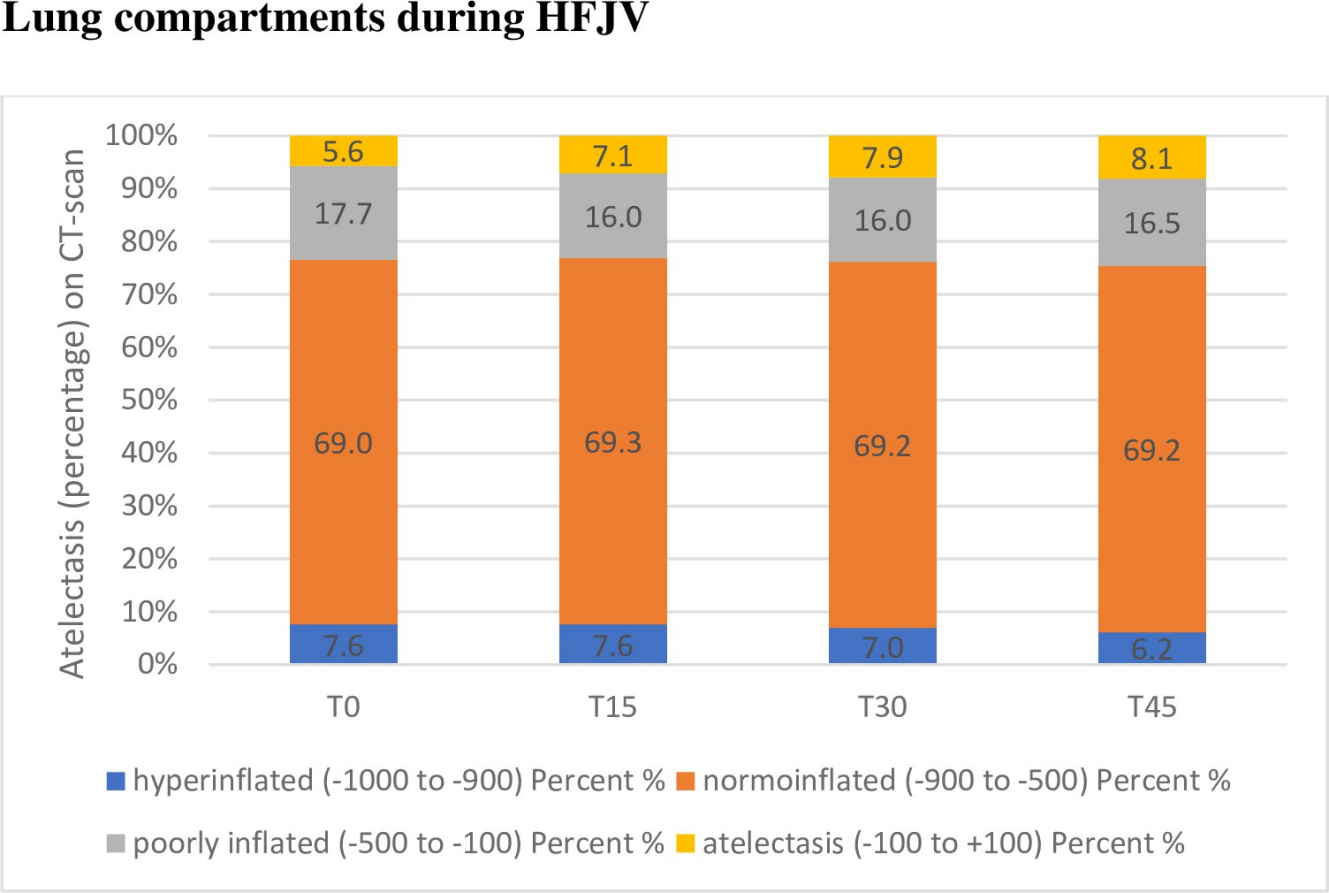
The four lung compartments, described as the mean percentage of the lung, during the time period studied. T = 0 being the first CT-scan after initiation of HFJV (high frequency jet ventilation) and thereafter CT-scans every 15 minutes. The normoinflated lung is unchanged during the whole time period studied (P = 0.8).

The atelectatic parts of the lungs, defined as HU -100 - +100, increased during the time studied, although only statistically significant after 30 and 45 minutes, p = 0.002 and p = 0.024 respectively, when compared to baseline. See Figs 1 and 2.

Other parts of the lungs, apart from the atelectatic areas, were also studied in the classical four lung compartment manner defined in the method section. The normo-inflated areas of the lungs were in domination and did not change significantly over time (p = 0.8), see Fig 2.

### Subgroup analysis of early CT-imaging and body mass index

**Fig 3:**
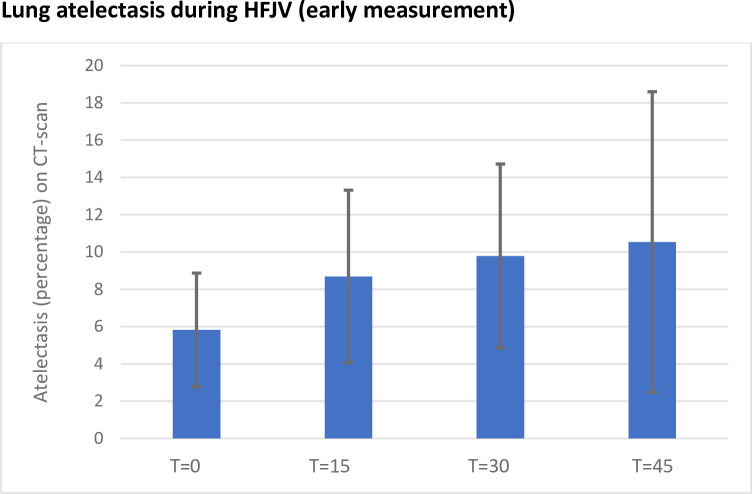
Mean atelectasis in percentage (HU -100-+100) at the four timepoints for the seven patients that started high frequency jet ventilation between 0–5 minutes before the first CT-image. T = 0 being the first CT-scan after initiation of HFJV (high frequency jet ventilation) and thereafter CT-scans every 15 minutes. Significant difference in the amount of atelectasis between groups was seen only at T30 when compared to baseline (p = 0.04).

A subgroup analysis was done for the seven patients where the first CT was taken within 5 minutes after the initiation of HFJV. Significant differences between groups were seen only at T30 when compared to baseline. See Fig 3.

**Fig 4:**
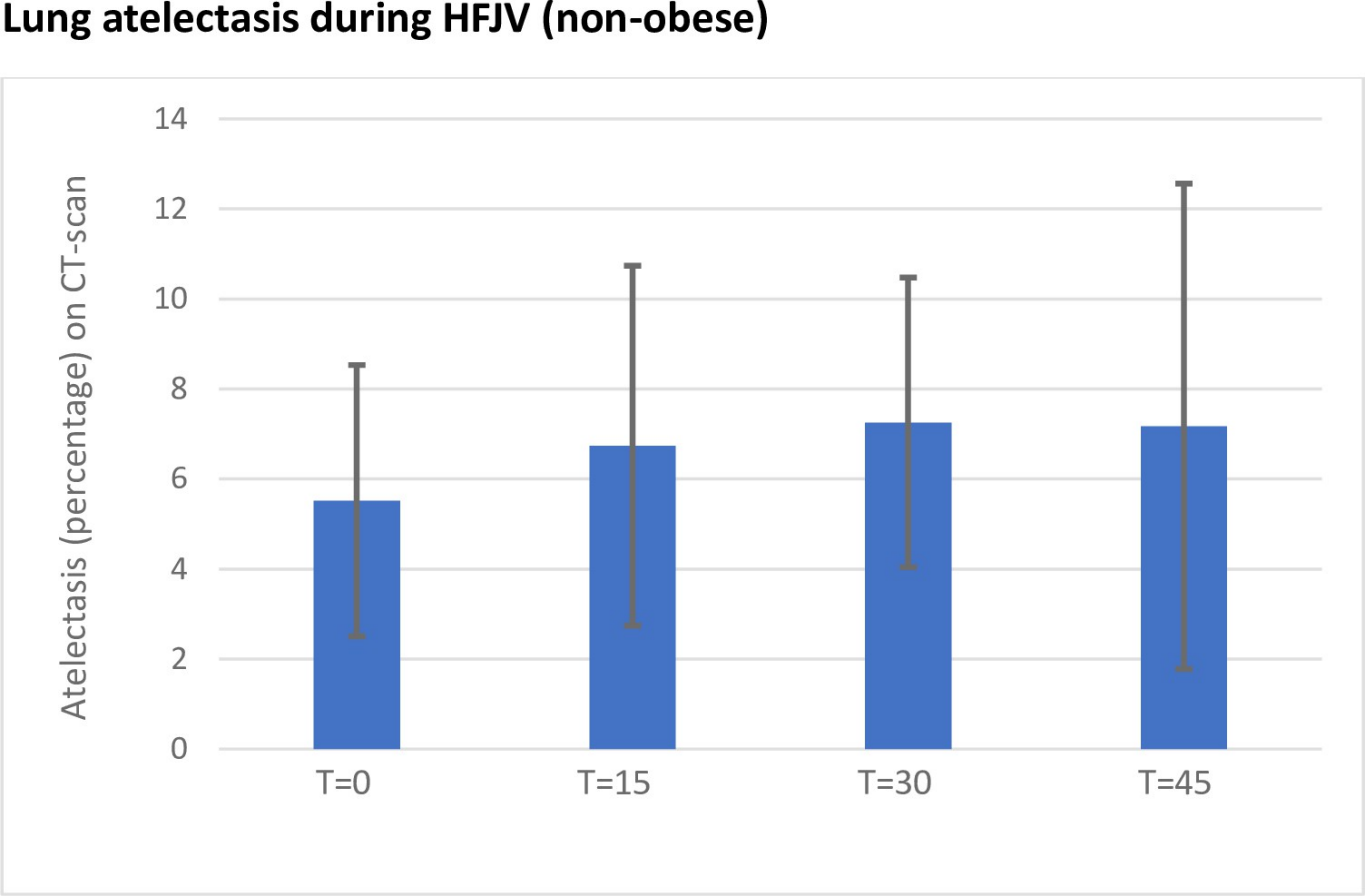
Mean atelectasis in percentage (HU -100-+100) at the four timepoints for the 15 patients with a body mass index<30. T = 0 being the first CT-scan after initiation of HFJV (high frequency jet ventilation) and thereafter CT-scans every 15 minutes. For this subgroup no statistical difference could be seen between the timepoints even though an initial visual trend is noted.

A subgroup analysis was also done for body mass index (BMI), where patients with a BMI<30 were analysed regarding the amount of atelectasis. Here, in this group of 15 patients, no significant difference could be seen between time-points. See Fig 4.

### Perioperative respiratory adverse events

No patient needed termination of HFJV due to elevated CO2. In one case tcCO2 rose to 10,6 kPa after the termination of the study protocol, HFJV was then converted to conventional ventilation. No event with low desaturation was noted intraoperatively. Lowest SpO2 was 93% in two patients who recovered and raised their SpO2 spontaneously during the surgery without any intervention.

**Fig 5:**
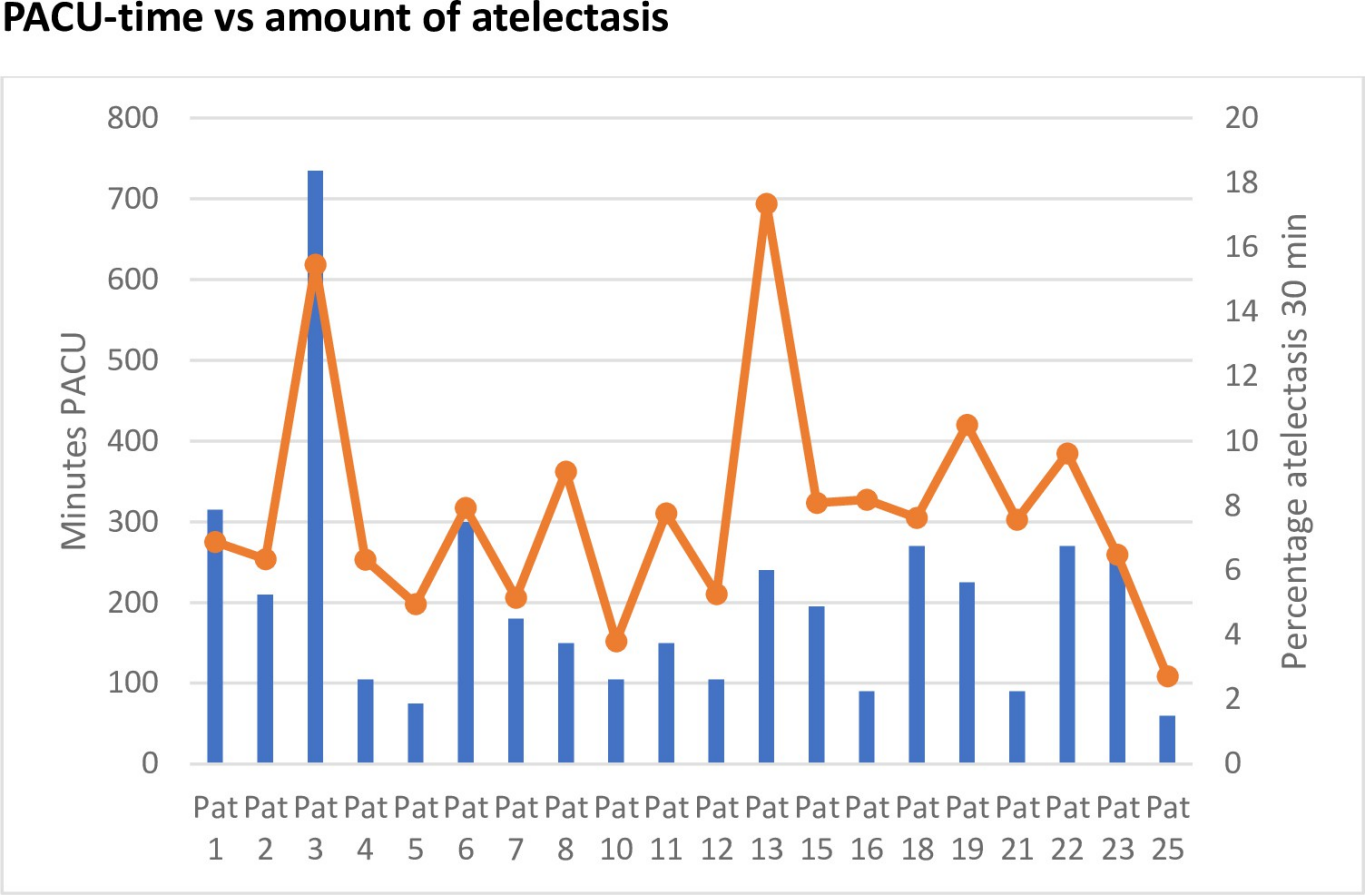
Time spent in the post anaesthesia care unit (minutes, left y-axis) and the amount of atelectasis at time 30 minutes (percentage, right y-axis) after start of HFJV (high frequency jet ventilation). Blue bars represent minutes in the PACU and orange dots represents amount of atelectasis.

The mean time in the PACU was 191 minutes. One patient was an extreme outlier with a PACU time of 735 minutes. This patient had a small pneumothorax perioperatively due to pleural puncture during the ablation procedure. It was left without intervention. In the PACU the patient experienced pain and received opioids, resulting in oversedation and need for prolonged continuous monitoring. After excluding this patient, the mean PACU time was 168 minutes. Only one patient had a saturation <90% (88%) postoperatively (total time in PACU 240 min). This patient also had the highest amount of atelectasis (17%) at T30 minutes and the second highest amount of atelectasis (19%) at T45 minutes. The highest amount of atelectasis at T45 minutes was the extreme outlier mentioned above. See Fig 5.

## Discussion

This study was performed to investigate the effects of HFJV on the quantity of lung atelectasis during general anaesthesia for percutaneous ablation of liver tumours. An increase of atelectasis during the 45 minutes study protocol was noted. There were however no significant changes in the normoinflated lung volumes, and poorly inflated areas decreased seemingly in relation to the increase in atelectatic volume. In a subgroup analysis with patients with a BMI < 30, no significant change in atelectasis was found.

In the present study, the initial lung CT-scan was performed with the patients anaesthetised, muscle relaxed and intubated in a supine position. The changes in atelectasis from awake to anaesthetised state could thus not be evaluated. The results must be assessed in the context of changes during HFJV only. During the procedure all patients had an inhaled oxygen fraction of 0.8. As this is an open system to room air, the alveolar fraction was probably lower, but still high in the context of the aims of the study. For safety reasons the high oxygen concentration was, at the time, a standard procedure, but it might have promoted the formation of the atelectasis by the absorption mechanism.

We are not aware of any studies investigating the effects of HFJV on lung compartments and atelectasis formation in this setting, and there is no control group in the present study. Therefore, comparison can only be made with previous studies where conventional ventilation was used with CT-imaging to quantify atelectasis. Brismar and colleagues [12] showed that after five minutes under general anaesthesia, with muscle relaxation, the area of atelectasis was greatest in the caudal parts of the lungs, 4,8% (SD 0,8). Another study by Cai and colleagues [17], investigated the effect of low tidal ventilation, LTV, (defined as 6 ml/kg) compared to traditional tidal ventilation, TTV, (10 ml/kg) in patients with no lung injury and how this would result in an increase in atelectasis formation. Sixteen patients were anesthetised and intubated, all with a body mass index < 25 and with an ASA physical status I or II. Atelectasis was seen in both groups. After intubation, the mean atelectatic area in the TTV group was 3.32% (SD 1.94) and in the LTV group the mean area was 4.19% (SD 2.31). At the end of surgery, the atelectatic area was 3.99% (SD 2.21) and 4.55% (SD 2.21) for the TTV and LTV respectively. As in the present study, Cai and colleagues used CT-images to quantify atelectatic area defined as HU -100 — +100, at one centimetre above the right diaphragm. Also, Eichenberger and colleagues [26] investigated atelectasis in a similar way with CT-imaging. They showed that morbidly obese patients have a significantly higher amount of atelectasis. After orotracheal intubation the morbidly obese patients had 7.6% of atelectasis versus 2.8% for the nonobese ones. Coussa and colleagues [27] showed similar results where morbidly obese patients, with or without PEEP of 10 cmH2O during preoxygenation and induction, after tracheal intubation, had 1.7% (SD1.3) and 10.4% (SD 4.8) of atelectasis respectively.

Data from these studies are comparable to the data in the precent study. Thus, the atelectasis formation during HFJV seems to be in the same magnitude as seen with conventional ventilation under general anaesthesia. Atelectasis increased over time and in agreement with previous studies on gas exchange, carbon dioxide increased while oxygenation was safely maintained [28]. It is noteworthy that the one patient with an oxygen desaturation during the recovery period, also had the greatest increase in atelectasis during HFJV.

Other alternatives to minimise organ movement during surgery have been explored. The use of THRIVE (trans nasal humidified rapid-insufflation ventilatory exchange) has recently been studied by Gustafsson and colleagues [29] and they could show that apnoeic ventilation using THRIVE was feasible for up to 30 minutes during laryngeal surgery in patients with mild to moderate systemic disease, i.e. ASA I and II. It is unclear if this is applicable to the patient population accepted for liver tumour ablation. Experiences from our institution with more than 1000 ablation sessions is that this patient group is usually elderely with an ASA score of II-IV and the operating time, depending on number of tumours being treated, is usually between 30 minutes to an hour, rarely up to four hours. Though this is a tempting method to use with its obvious advantages in reducing respiratory related movements of upper abdominal organs, there are also clear limitations.

### Limitations

The present study is limited by the number of patients and the lack of a control group with conventional ventilation. In stereotactic, CT-guided ablations, a control group with conventional ventilation is not possible due to unacceptable breathing related movement of the liver. We do not have a baseline CT-scan, neither before start of anaesthesia nor before start of HFJV. The first CT-scan analysed was taken several minutes after initiation of HFJV. As it is known that the formation of atelectasis starts immediately after induction [11], the results of this study might have been somewhat different if the scans had been taken immediately after the start of HFJV.

Before the first CT-scan was performed, all patients received intravenous contrast as a part of the routine procedure for this type of surgery. Contrast media in a CT-scan partly overlaps the HU of that of atelectasis. This means that the first CT-scan could contain a falsely high atelectatic area. Still, this is the lowest mean value of atelectasis during the time studied. Moreover, our study follows the atelectasis formation during the time span of the intervention: being the administration of contrast media consistent across the studied patients, its effects were deemed not influencing the global result of the present study.

In conclusion, the use of HFJV during general anaesthesia in patients undergoing liver ablation in supine position causes an increase in atelectasis formation while poorly inflated volume decrease. The normally inflated volume is not affected. The amount of atelectasis found seems to be in the same range as has previously been found during general anaesthesia with conventional ventilation. The benefits in the use of HFJV in reducing ventilation-related tissue movements, reducing risks associated with high precision liver and pancreas ablations, by far outweigh its risks.

## Supporting information

S1 Data (XLSX)

S1 Protocol Study protocol. (DOCX)
