# Supplementary material for: Quantitative assessment of atelectasis formation under high frequency jet ventilation during liver tumour ablation–A computer tomography study
Source: PLoS One. 2023 Apr 3;18(4):e0282724. doi: 10.1371/journal.pone.0282724 (PMC10069764; doi:10.1371/journal.pone.0282724)
Supplement: S1 Protocol — (DOCX) [file pone.0282724.s002.docx]

**Study protocol**

**Quantitative Assessment of Atelectasis Formation under High Frequency Jet Ventilation during Liver Tumour Ablation – a Computer Tomography Study.**

Principal investigator Karolina Galmén

In collaboration with Jan G Jakobsson, Gaetano Perchiazzi, Jacob Freedman and Piotr Harbut

**Background**

Thermo-ablation of liver tumours (metastasis or primary tumours) has been performed at Danderyd Hospital for several years. As the navigation of the ablation needle is performed with non-real time CT-scans, it is of great importance that the liver is as immobilized as possible. The breathing related liver movements can be minimized by using high frequency jet ventilation (HFJV)(1)(2). HFJV have been used in clinical practice since the 1970’s, mostly in the area of ear-, nose- and throat surgery with the purpose not to interfere with the surgical field(3). During the past years HFJV has been used for the purpose to immobilize organs (mostly liver, lung, pancreas, and kidney). This leads to increased surgical precision and hopefully better outcome for the patients(4)(1). Supine position and general anaesthesia are known to produce changes in aeration of the lung, increasing formation of atelectasis. How the lung is affected during HFJV when used during liver ablation with patients in a supine position in an X-ray laboratory is not studied.

**Aim**

The aim of the present study is to investigate, by repeatedly CT-images performed during stereotactic ablation, how the lung aeration change and formation of atelectasis during HFJV.

**Method**

Following informed consent, 25 consecutive patients scheduled for stereotactic ablation of liver tumours will be recruited into the study. Exclusion criteria are patients below 50 years of age and patients with severe lung disease.

Primary outcome in this study is to measure the amount of atelectasis over time during HFJV. A secondary aim was to assess the frequency and extent of perioperative respiratory adverse events.

*Study procedure*

- An arterial line is placed before or after induction of anaesthesia depending on clinical indication and ASA-score.
- Routine monitoring including transcutaneous CO2 will be started. Baseline values are documented.
- Induction, intubation and recruitment according to the guidelines of the HFJV is initiated with standard settings: DP 1,2-1,4 bar, frequency 220/min, O_2_ 80%, humidification level 4, I:D 40%.
- IV contrast is given.
- CT-liver and the first CT-scan of the liver is performed.
- Hereafter a new CT of the lung is performed every 15 minutes with a total of 4 images.
- The HFJV is terminated at the end of the ablation procedure
- Patients will be extubated in accordance to clinical routines and transferred to the recovery room
- Recovery room observations will follow departmental routines and patients will be discharged to the general floor in accordance to department practice
- Study ends at discharge from the recovery room

*Method of anaesthesia*

All patients will have total intravenous anaesthesia with [propofol](https://www-sciencedirect-com.sll.idm.oclc.org/topics/medicine-and-dentistry/propofol) (*Propofol-Lipuro®, B. Braun Melsungen AG, Melsungen, Germany*) and [remifentanil](https://www-sciencedirect-com.sll.idm.oclc.org/topics/medicine-and-dentistry/remifentanil) (*Ultiva®, GlaxoSmithKline AB, Solna, Sweden*). Neuromuscular block will be established by incremental doses of rocuronium (*Esmeron®, MSD, Haarlem, Netherlands*). Intubation will be performed with an endotracheal tube, a size 9 for men and size 8 for women.

*Monitoring*

All patients will be monitored in accordance with department routines with standard ECG, SpO2, invasive blood pressure and trans-cutaneous CO2 (*Radiometer TCM5, Triolab AB, Mölndal, Sweden*).

*HFJV*

High frequency jet ventilation will be commenced by disconnecting the endotracheal tube from the conventional ventilator and inserting a thin jet cannula (*Laserjet 40, double lumen jet catheter acc Biro, Acutronic Medical System AG, Hirzel, Germany*) through the endotracheal tube. A Monsoon III ventilator will be used (*Acutronic, Switzerland*). Fixed settings for driving pressure (DP) 1,2-1,4 bar, oxygen supplementation FiO2 0,8, frequency 220/min and I/E (Inspiration duration ID) ratio 40% will be applied.

*CT-scans*

CT-scans of the lung will be performed at four different time points during surgery with 15 minutes’ interval. The first CT will be performed at the time when HFJV is initiated. The lung programme in the CT-machine software will be specifically set up for this study to get as low radiation dose as possible for acceptable quality for analysis. The CT-images will be a volume of 10 cm at 50 mAs and 120 kV, with a slice thickness of 0,5 mm. In some cases, the time for lung scanning (every 15’) will coincided with the treatment scanning of the liver and lung bases for the ablation procedure, with exposure at 140 mA and 120 kV, the slice thickness being 1 mm. All scans will be analysed at the level one cm above the right diaphragm.

*Assessment of CT-scans*

In the CT scans, lung parenchyma will manually outlined by tracing the border between the pleura and the surrounding tissues by an experienced radiologist. The CT images will be further analysed using scripts for the Image Processing Toolbox for MatLab R2020 (*MatLab, The MathWorks, Natick, MA, USA*), purposely written by one of the authors (G.P.). By this way we will assess aeration in accordance to previous convention (5) (6), four lung compartments: hyperinflated (HU between − 1000 and − 900), normoinflated (HU between − 900 and − 500), hypoinflated (HU between − 500 and − 100) and non-inflated or atelectatic (HU between − 100 and + 100). The extension of each lung compartment will be expressed as percentage of the total lung area in each scan.

*Collection of data*

All study data will be collected in an individual case record form. Each patient will receive a

study code. Code list will be stored on the hospital secure IT-platform. Coded study data will further be aggregated into a standard XL-file and analysed with SigmaPlot (*version 14, Software Inc, San Jose, California, USA*).

*Study conduct approvals*

Ethical review board, X-ray safety review board and university approvals will be sought and collected before start of study. Inform consent leaflet and consent form will follow the guide from the National Swedish Ethical Review Authority. The study will be registered in Clinical Trial Gov.

**References**

1. Biro P, Spahn DR, Pfammatter T. High-frequency jet ventilation for minimizing breathing-related liver motion during percutaneous radiofrequency ablation of multiple hepatic tumours. BJA Br J Anaesth [Internet]. 2009 May 1;102(5):650–3. Available from: http://dx.doi.org/10.1093/bja/aep051

2. Raiten J, Elkassabany N, Mandel JE. The use of high-frequency jet ventilation for out of operating room anesthesia. Curr Opin Anaesthesiol. 2012 Aug;25(4):482–5.

3. Bohn D. The history of high-frequency ventilation. Respir Care Clin N Am. 2001 Dec;7(4):535–48.

4. Engstrand J, Toporek G, Harbut P, Jonas E, Nilsson H, Freedman J. Stereotactic CT-Guided Percutaneous Microwave Ablation of Liver Tumors With the Use of High-Frequency Jet Ventilation: An Accuracy and Procedural Safety Study. AJR Am J Roentgenol. 2017 Jan;208(1):193–200.

5. Gattinoni L, Pesenti A, Bombino M, Baglioni S, Rivolta M, Rossi F, et al. Relationships between lung computed tomographic density, gas exchange, and PEEP in acute respiratory failure. Anesthesiology. 1988 Dec;69(6):824–32.

6. Vieira SR, Puybasset L, Richecoeur J, Lu Q, Cluzel P, Gusman PB, et al. A lung computed tomographic assessment of positive end-expiratory pressure-induced lung overdistension. Am J Respir Crit Care Med. 1998 Nov;158(5 Pt 1):1571–7.
